# IL-33 Mediated Inflammation in Chronic Respiratory Diseases—Understanding the Role of the Member of IL-1 Superfamily

**DOI:** 10.3389/fimmu.2019.00692

**Published:** 2019-04-16

**Authors:** Agata Gabryelska, Piotr Kuna, Adam Antczak, Piotr Białasiewicz, Michał Panek

**Affiliations:** ^1^Department of Sleep Medicine and Metabolic Disorders, Medical University of Lodz, Łódz, Poland; ^2^Department of Internal Medicine, Asthma and Allergy, Medical University of Lodz, Łódz, Poland; ^3^Department of General and Oncological Pulmonology, Medical University of Lodz, Łódz, Poland

**Keywords:** asthma, COPD—Chronic obstructive pulmonary disease, OSA (Obstructive sleep apnea), IL-33, inflammation

## Abstract

Interleukin 33 (IL-33) is an alarmin cytokine from the IL-1 family. IL-33 is localized in the nucleus and acts there as a gene regulator. Following injury, stress or cell death, it is released from the nucleus, and exerts its pro-inflammatory biological functions via the transmembrane form of the ST2 receptor, which is present mainly as attached to immune cells. In recent years, IL-33 became a focus of many studies due to its possible role in inflammatory disorders. Among respiratory disorders, the contribution of IL-33 to the development of asthma, in particular, has been most identified. Increased level of IL-33 in lung epithelial cells and blood serum has been observed in asthma patients. The IL-33/ST2 interaction activated the Th2 mediated immune response and further production of many pro-inflammatory cytokines. Single nucleotide polymorphisms in the IL-33 gene cause a predisposition to the development of asthma. Similarly, in chronic pulmonary obstructive disease (COPD), both increased expression of IL-33 and the ST2 receptor has been observed. Interestingly, cigarette smoke, a key inducer of COPD, not only activates IL-33 production by epithelial and endothelial cells, but also induces the expression of IL-33 in peripheral blood mononuclear cells. Knowledge regarding its contribution in other respiratory disorders, such as obstructive sleep apnea, remains greatly limited. Recently it was shown that IL-33 is one of the inflammatory mediators by which levels in blood serum are increased in OSA patients, compared to healthy control patients. This mini review summarizes current knowledge on IL-33 involvement in chosen chronic respiratory disorders and proposes this interleukin as a possible link in the pathogenesis of these diseases.

## Introduction

The inteleukin-1 (IL-1) superfamily of cytokines plays a pivotal role in both innate and adaptive immunity by regulating host defense, inflammation and injury (1, 2). Similarly to the transforming growth factor, the Smad (TGF-Smad) superfamily, members of the IL-1 superfamily are associated with chronic pulmonary disorders and fibrosis (3).The IL-1 superfamily includes 11 members: IL-1α, IL-1β, IL-1 receptor antagonist (IL-1Ra), IL-18, IL-33, IL-36α, IL-36β, IL-36γ, IL-36Ra, IL-37, and IL-38 (4, 5). All family members are characterized by a similar gene structure, in some cases having identical intron positioning and amino acids sequences, enabling folding of the protein into the three dimensional structure of a 12-stranded-β-barrel (6). Furthermore, all genes encoding members of IL-1 other than IL-18 and IL-33 occupy approximately a 400 kB interval on chromosome 2 (7). All the biologically active cytokines of the family are extracellular molecules, while their precursors are primarily intracellular with an exception of IL-1Ra, which encodes classical signal peptide. IL-1β and IL-18 achieve biological activity only after their precursors are cleavaged from the pro-domain by inflammasome. In the case of IL-36 α, β, γ, IL-36Ra, and IL-33, the full-length molecules already possess biological activity, however, following the cleavage of the N-terminus they obtain full biological potency (8, 9). Origin and function is not the only characteristic feature uniting the cytokines within IL-1 super family, as they also relate to each other by transduction pathways and receptor structure, all consisting of three Ig-like domains and Toll/IL-1R domain (1).

## IL-33

IL-33, one of the most recently discovered members of IL-1 superfamily, is an alarmin cytokine promoting inflammatory responses (5). Originally it was known as a nuclear factor from high endothelium venules (HF-HEV) (10), and only later has its cytokine activity been defined (11). It mainly expresses itself through epithelial and endothelial cells, fibroblasts-like cell and myofibroblasts (10–13).

Differently to most of the members of IL-1 superfamily, the gene encoding IL-33 is localized on the short arm of chromosome 9, at 9p41.1 (14). The gene is comprised of 8 exons, poses interferon stimulated response element (ISRE), and several gamma interferon activation sites (GAS) in the promotor area (15). It is constitutively expressed in its pro form of 270 amino acids (full length IL-33; f-IL 33), with its molecular mass of 30 kD being stored in the nucleus of cells where it acts as a nuclear regulator (10, 11, 16). This function is facilitated by its N-terminal nuclear domain, which contains a chromatin binding motif that binds to histones (17, 18). Two other domains building up IL-33 are the IL-1-like cytokine domain, and the central domain (11, 19). IL-33 has been identified as a dual function cytokine, also possessing a full bioactivity as an alarmin upon its release from a cell (20). Furthermore, f-IL-33 serves as a substrate for serine proteases of neutrophils and mast cells. It results in shorter forms of a peptide comprised of IL-1 like domain and part of central domain, forming a mature form of IL-33 (m-IL-33). It is estimated that bioactivity of m-IL-33 increases ~10–30 times compared to f-IL-33 (8, 19). During apoptosis caspase-3 and caspase-7 cleave f-IL-33, resulting in peptide, which lacks biological activity (9). It has been shown that the expression of IL-33 can be increased following the exposure to cigarette smoke (21). Other factors that can induce the same effect include viral infection and exposure to allergens such as pollen, chitin, fungi, or *Alternaria* spp. (22–28). The main mechanism in which the activity of IL-33 is limited is sequestration by soluble isoform of its suppression of tumorigenicity 2 (sST2) receptor that acts as a decoy, inhibiting the expansion of IL-33 mediated inflammation (29–31). Regulation of IL-33 activity also includes oxidation of cysteine residues following extracellular release and formation of disulphide bridges, resulting in the inactivation of IL-33 (24).

**Figure 1:**
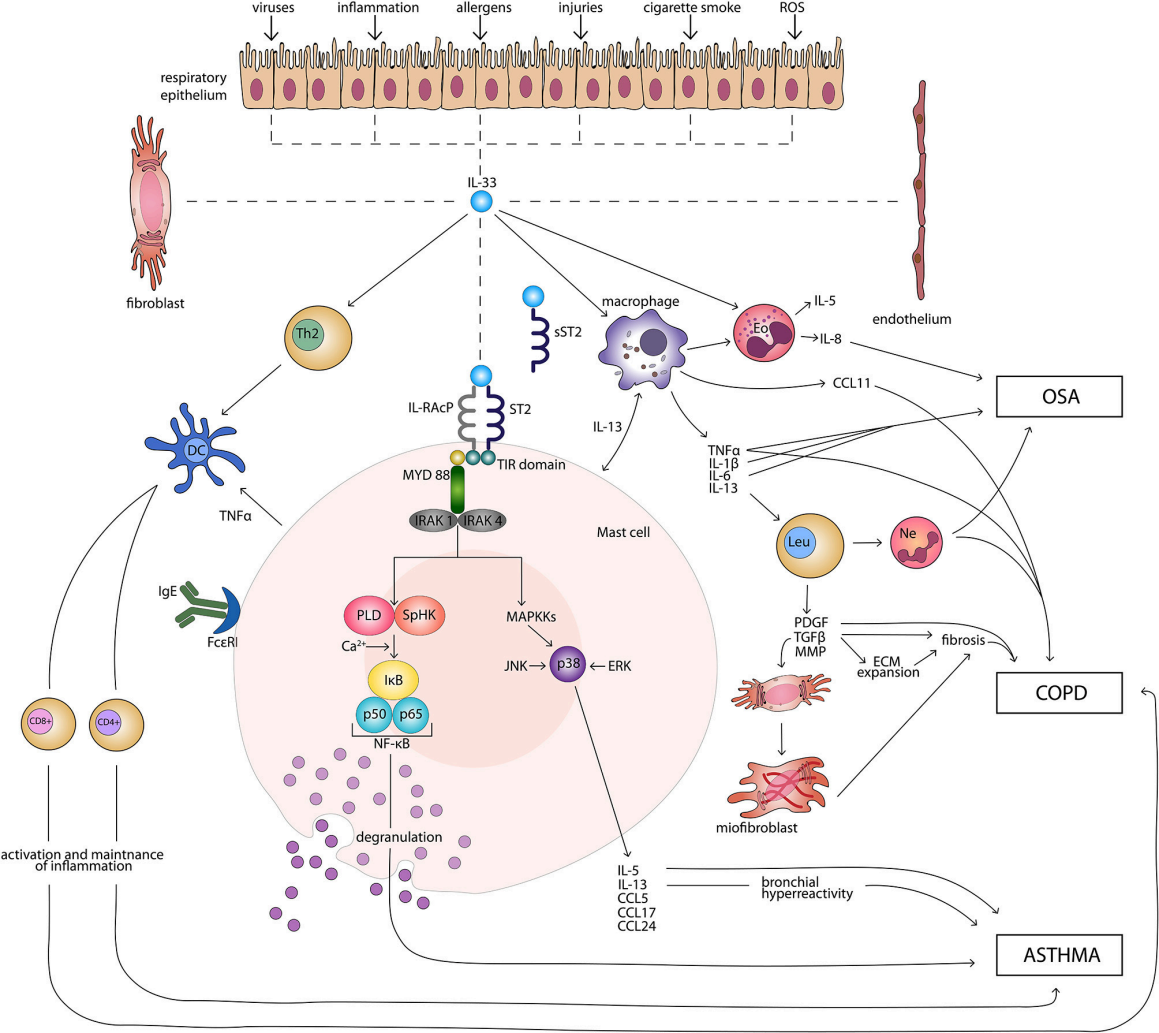
Main directions of IL-33 molecular function in induction and maintenance of chronic respiratory disorders: asthma, COPD, and OSA. CD4+, CD4+ T cell; CD8+, CD8+ T cell; CCL, CC-chemokine ligand; COPD, chronic obstructive pulmonary disease; DC, dendritic cell; ECM, extracellular matrix; Eo, eosinophil; ERK, extracellular signal-regulated kinase; FcεRI, high-affinity receptor for the Fc region of immunoglobulin E; IgE, immunoglobulin E; IκB, inhibitor of κB; IL, interleukin; IL-RAcP, interleukin 1 receptor accessory protein; IRAK, interleukin-1 receptor associated kinase; JNK, c-Jun N-terminal kinases; Leu, leucocyte; MAPKKs, mitogen-activated protein kinase kinases; MMP, matrix metalloproteinases; MYD 88, myeloid differentiation primary response protein 88; Ne, neutrophil; NF-κB, nuclear factor κB; OSA, obstructive sleep apnea; PDGF, platelet-derived growth factor; PLD, phospholipase D; ROS, reactive oxygen species; SpHK, sphingosine kinase; ST2, suppression of tumorigenicity 2 transmembrane receptor; sST2, suppression of tumorigenicity 2 soluble receptor; Th2, T helper 2 cell; TIR, Toll-like/interleukin 1receptor; TGF-β, transforming growth factor β; TNFα, tumor necrosis factor α.

IL-33 signaling pathway begins with binding of the cytokine to the ST2 receptor. This transmembrane form of the receptor is generated by the same mRNA as the sST2 and the distinction is made through a different promoter and alternative splicing of the transcript (32, 33). Following the attachment of the protein to ST2, the corrector IL-1 receptor accessory protein IL-1RAcP is recruited and further heterodimeric signaling complex is formed, which involves myeloid differentiation primary response protein 88 (MYD88), IL-1R-assosiated kinase 1, and 4 (IRAK1, IRAK4), as well as tumor necrosis factor (TNF) receptor-assosiatedreceptor-6 (TRAF6) (11, 34, 35). This results in nuclear factor κB (NFκB) transcription and activation of mitogen-activated protein kinases (MAPK), including c-Jun N-terminal kinases (JNK) and p38 (11), which then in a signaling pathway drive processes such as proliferation, cell survival, cytokine secretion (IL-4, IL-5, and IL-13) and amphiregulin (AREG) expression (36–38). Only ST2^+^ cells are affected by IL-33 stimulation. The type of response differs depending on the type of stimulated cell, as T helper 2 (Th2) cells respond by the secretion of Il-5 and IL-13 (11, 39), while mast cells release a different profile of cytokines, including IL-4, IL-5, and IL-6 (40). On the other hand, neutrophils following IL-33 stimulation migrate through chemotaxis (41). In the case of eosinophils, their response consists of degranulation and reactive oxygen species (ROS) production (36, 42, 43), while macrophages in response to IL-33 develop an alternatively activated phenotype (44, 45). IL-33 has also been shown to activate NFκB and p38 in regulatory lymphocytes T (Tregs), which further leads to the expansion of ST2^+^ Tregs (46). Additionally, IL-33 stimulates TGF-β-mediated Treg differentiation (47). Activation of Tregs through MYD88 has been observed only in the IL-33/ST2 independent manner (48). It remains unknown if Tregs function can be affected in IL-33/ST2- MYD88 dependent pathways (49). Main directions of IL-33 molecular function in induction and maintenance of chronic respiratory disorders: asthma, COPD, and OSA are shown in Figure 1.

## Asthma

Among chronic respiratory disorders, the IL-33 contribution to disease development has been furthest identified in bronchial asthma. Increased levels of IL-33 in lung epithelial cells and blood serum has been observed in asthma patients. In several genome wide association (GWA) studies it has been established that both genes encoding IL-33 and its receptor IL1RL1 are susceptibility loci for asthma (50–55). The GWA studies have identified single nucleotide polymorphisms (SNPs) associated with asthma in general, as well as in different clinical phenotypes (56–59). A significant connection was identified, for example, between SNP of IL-33—rs3939286—and atopic asthma, as well as eosinophil blood count (60). Similar observations were made for multiple SNPs in the gene encoding ST2 receptor. One example of the SNP of IL1RL1 that predisposes to both atopic and non-atopic asthma and is connected with eosinophilia, causing an increased level of serum IgE and airway hyperresponsiveness, is rs950880 for non-Islandic population (in Islandic population it is rs1420101) (60, 61). The SNP (rs10204137) in the Toll-like/IL-1 (TIR) domain of ST2 can enhance the IL-33/ST2L pathway through further cascade (62). What is very interesting, and highlights the role of IL-33 in the development of asthma, is a rare loss of function mutation (rs146587587-C), in which premature STOP codon causes truncation of the last 66 amino acids of the cytokine. This results in a reduced eosinophil blood count and protection against asthma (63).

Several mechanisms in which IL-33 participates in the development of asthma have been described.

It has been shown that IL-33 promotes lung fibrosis and bronchial remodeling, causing further advancement of asthma (64, 65). IL-33 participates in the process of fibrosis by directly activating cells to produce the profibrotic factors as well as by inducing inflammation. Following damage, cells secrete IL-33, which activates a response from cells that possess IL1RL1 receptor. In a Th2 mediated immune response, innate lymphoid cells (ILC) and Th2 cells produce pro-inflammatory cytokines (IL-4, IL-5, and IL-13) (66), which have been identified as important mediators of fibrosis (67, 68). IL-4 and IL-13 are responsible for recruitment of basophils and eosinophils that cause differentiation of fibroblasts to myofibroblasts and collagen deposition (69). Same cytokines induce macrophage polarization to an alternatively activated profibrotic phenotype (44). IL-5 secreted by Th2 cells also takes part in the recruitment and activation of eosinophils, which are the source of profibrotic factors such as TGF-β, PDGF, and IL-13 (70). Interestingly, it has been shown that IL-33 can induce IL-5-producing T cells and promote airway inflammation independent of IL-4, which was shown in IL-4 deficient mice (45).The increased tissue fibrosis can be enhanced by interaction between major histocompatibility complex II (MHCII) of ILCs and Th2 cell receptor (TCR), which activates Th2 cells. Additionally, IL-33 itself recruits basophil, eosinophils, and mast cells to the local inflammatory site and activities them into the production of profibrotic factors (71). Moreover, it was observed that IL-33 directly promotes fibroblasts to produce fibronectin 1 and collagen type 1, factors involved in airway remodeling (65). Interestingly, it has been shown that IL-33, similarly to TGF-β, not only activates the fibroblasts, but also promotes epithelial-to-mesenchymal transition (EMT), which is associated with overexpression of profibrotic markers (72, 73). IL-33 can also contribute to the development of allergen driven airway inflammation by dysregulating Treg and impairing immunologic tolerance to inhaled antigens. Treg cells in lungs exposed to IL-33 not only upregulate their expression of Th2 transcription factor GATA binding protein 3 (GATA3) and ST2, but also enhance production of type 2 cytokines. In effect, Tregs lose their ability to supress effector T cells resulting in development of airway inflammation (74).

Bronchial hyperreactivity is typical in the course of asthma and IL-33 has been identified as inducing this process. It has been shown that through the activation of IL-5 and IL-13 secretion, IL-33 promotes airway hyperresponsiveness (75). It has been suggested that this is dependent on smooth muscle cells (76, 77). Other possible mechanisms involve irritation caused by secretion of serotonin by mast cells following the IL-33 activation (78). Feedback circuit involving both IL-33 and ILCs responsible for bronchial hyperactivity and persistence of asthma has been suggested based on the animal model of the disease (79).

## Chronic Obstructive Pulmonary Disease

Chronic pulmonary obstructive disease (COPD), is a progressive inflammatory condition, in which an increased expression of IL-33 and the ST2 receptor, similarly to asthma, has been observed (27, 80). Cigarette smoke has been identified as the key inducer of COPD. In the mouse model of cigarette smoke-induced COPD, both IL-33 and the ST2 receptor expression was increased in lungs (27, 81, 82). Inhibition of cigarette-induced pathogenic changes in lungs have been observed following introduction of anti-IL-33 antibody (81), which suggests an important role of IL-33 in the pathogenesis of COPD. Furthermore, mice treated with IL-33 developed histopathological changes in the lungs such as lining hypertrophy, goblet cell hypertrophy, and mucus hypersecretion, which are typical for COPD patients (11). Interestingly, cigarette smoke not only activates IL-33 production by epithelial and endothelial cells, but also induces the expression of IL-33 in peripheral blood mononuclear cells (PBMC) (82) and peripheral blood lymphocytes (PBL) (80). This systemic response trough activation of the immune system might enhance processes involved in the development of COPD. Additionally, cigarette smoke alters the distribution of the expression of IL-33 receptor ST2 (21). Decreased expression of the receptor was observed on ILC2s, while macrophages and natural killer cells presented elevated expression of this receptor. This change significantly amplified type I proinflammatory response within the lungs, exaggerating exacerbations of COPD in the course of infections (21). Infections play an important role in progression of COPD and greatly increase the risk of death. Lipopolysaccharide (LPS) has been shown to enhance IL-33 expression and release not only in epithelial cells, but also in PBMC and PBL (80). This suggests that IL-33 is an important mediator of immunological response during inflammations and exacerbations in the course of COPD. It has been widely established that COPD patients suffer from chronic inflammation, which leads to alveolar disruption. It has been shown that IL-33 induces IL-6 and IL-8 production and release in lung epithelial and endothelial cells (82, 83). The same two interleukins have been observed to be increased in bronchoalveolar lavage (BAL) and the lungs of COPD patients, compared to healthy controls (84–86). This causes an influx of neutrophils to the lungs which, through the secretion of elastases and proteases, cause lung tissue damage, further resulting in lung fibrosis and decreased lung function (87). Additionally, it has been shown that IL-33 is involved in the development of eosinophilic airway inflammation in non-atopic COPD patients (88). Moreover, IL-33 plays a part in mucus production, advancing the inflammatory process and decreasing respiratory capacity of COPD patients (27). What is more, IL-33 increases vascular endothelial permeability, which further intensifies inflammatory effect (89). It has been shown that IL-33 can directly contribute to lung tissue damage trough triggering cortactin degradation mediated apoptosis in alveolar epithelial cells (90).

## Obstructive Sleep Apnea

Obstructive sleep apnea (OSA) is a chronic condition characterized by recurrent pauses in breathing during sleep caused by collapse of the upper airways. The disorder is highly associated with chronic low-grade systemic inflammation. The most prominent inflammatory mediators present in OSA patients include IL-1, IL-6, and CRP (91). Literature concerning IL-33 in OSA patients is greatly limited. In recent studies, it has been observed that IL-33 is one of the inflammatory mediators causing levels in blood serum to be increased, in comparison to healthy controls (92). Interestingly, increased IL-33 levels have also been shown in the saliva of OSA patients (93). This emphasizes the contribution of IL-33 to systemic inflammation in this group of patients. Substantial obesity is a well-known risk factor for OSA (94). Excessive amounts of central adipose tissue also contributes to levels of systemic inflammation among OSA (95). Adipocytes have been identified to possess ability to produce IL-33 (96), therefore adipose tissue in OSA patients offers an extensive source of IL-33. Additionally, intermittent hypoxia contributes to systemic inflammation (97). It can possibly affect the IL-33 production too, however the mechanisms of this relationship are not known yet. As described before, IL-33 highly contributes to the development of chronic inflammatory diseases such as asthma and COPD. Several studies have shown increased prevalence of these diseases among OSA patients compared to the general population (98–100). This suggests that OSA patients are vulnerable to the development of comorbidities of inflammatory etiology which are not only limited to respiratory disorders but also others, such as diabetes or psoriasis (97, 101–103). It has been shown that OSA with severe co-morbid asthma intensifies airway remodeling and is associated with more frequent exacerbations (104, 105). Even though IL-33 has not been investigated as a mediator in a group suffering from these two co-morbid diseases, it can be hypothesized that this alarmin enhances airway remodeling and fibrosis occurring in the course of asthma due to its increased level caused by co-morbid OSA. Similar observations have been made regarding co-morbid OSA and COPD, as the BAL fluid of patients with the overlapping syndromes showed a significantly increased proportion of neutrophils, higher TNFα concentrations, and IL-8 levels than that of COPD. Co-morbid OSA exacerbated the course of COPD (106). Yet again IL-33 was not investigated in the study. However, it may again be assumed that systemic inflammation present in OSA with increased IL-33 levels intensifies processes involved in the development and progression of COPD.

## IL-33 as a Potential Therapeutic Target

The murine models of both asthma and COPD suggest that IL-33 and the ST2 receptor might be prominent new therapeutic targets for these chronic inflammatory respiratory diseases. In a murine model of asthma, the use of anti-IL-33 antibody reduced Th2 cytokines production by ILC2 (107), while the antibody blocking ST2 receptor caused a decrease of interleukin 4 expression in the lungs of mice undergoing ovalbumin challenge and extenuated airway hyperresponsiveness (108). In another study a decrease in eosinophil count in BAL fluid and reduced airway hyperresponsiveness to methacholine was observed following anti-IL-33 antibody and sST2 receptors treatment (109). It has been shown, *in vitro*, that pre-treatment with anti-ST2 antibody supressed the production of fibronectin 1 and type I collagen by human lung fibroblasts (65). Anti-IL-33 antibody has also been shown to significantly inhibit cigarette smoke induced lung inflammation as, following the treatment, neutrophil and macrophage infiltration along with cytokines (IL-1β, TNF, and IL-17) expression decreased (81).

Several clinical trials targeting either IL-33 or its receptor in asthma and COPD are ongoing at the moment. In COPD two trials concerning IL-33/ST2 have reached phase 2. One investigates the effects of anti-IL-33 monoclonal antibody compared with placebo, on the annualized rate of moderate-to-severe acute exacerbations of COPD over up to 52 weeks of treatment (ClinicalTrials.gov: NCT03546907). The second evaluates the efficacy of anti-ST2 antibody vs. placebo on the frequency of moderate-to-severe exacerbations of COPD (ClinicalTrials.gov: NCT03615040). At phase 2 of the asthma clinical trial, the anti-IL-33 receptor monoclonal antibody is investigated in subjects with moderately severe asthma, and is compared to the placebo, fluticasone propionate/salmeterol combination and fluticasone propionate (ClinicalTrials.gov: NCT03207243). Another clinical trial concerning anti-IL-33 antibody in asthma patients is in phase 1 and compares it to the placebo Dupilumab and fluticasone propionate (ClinicalTrials.gov: NCT03112577) (Table 1).

**Table 1:** Summary of ongoing clinical trials targeting IL-33 pathway in COPD and asthma.

**Condition**	**Intervention**	**ClinicalTrials.gov identifier**	**Comparison**	**Phase**
COPD	Anti-IL-33 monoclonal antibody	NCT03546907	Placebo	2
COPD	Anti-ST2 antibody	NCT03615040	Placebo	2
Asthma	Anti-IL-33 receptor monoclonal antibody	NCT03207243	Placebo/fluticasone propionate with salmeterol/ fluticasone propionate	2
Asthma	Anti-IL-33 monoclonal antibody	NCT03112577	Placebo/Dupilumab/ anti-IL-33 antibody with Dupilumab/fluticasone propionate	1

## Conclusion

Analysis of the pleiotropic effects of IL-33 on multiple immunological cells (macrophages, mastocytes), as well as neurological cells of medulla oblongata, dorsal root ganglion, antigen-induced arthritis system, carrageen, and formalin, shows that this alarmin plays curtail, yet not fully known role in mediating inflammation, especially in chronic inflammatory pulmonary diseases such as asthma, COPD, and OSA. Taken into consideration the engagement in this process, in particular of mastocytes and their secretion of CXCL2, 4, 8, and other cytokines, there is no doubt regarding the etiopathogenic role of IL-33 in the development of asthma in response to various stimuli damaging bronchial epithelial cells. Additionally, IL-33 intensifies recruitment of eosinophils, macrophages, and Th2 lymphocytes, which again confirms its inflammatory role. Particularly important in the context of aggravation of chronic inflammation and progression of respiratory disease is IL-33 mediated influx of neutrophils and macrophages. As these cells secrete IL-1β, TNF-α, and release proteases (elastases, metalloproteinases, cathepsins, and proteinases), it is only a logical consequence that IL-33 is involved in development of lung emphysema and chronic bronchial inflammation. It is worth mentioning that IL-33 expression is enhanced following exposure to cigarette smoke, which correlates with increased number of lymphocytes CD8+, resulting in the release of perforin and granzymes. However, surprisingly, this model of COPD development was only confirmed during experiments on mice and was observed following the introduction of anti-IL-33 antibody. Among discussed respiratory disorders, the role of IL-33 in OSA is the least known. The alarmin indirectly increases the expression of IL-1, IL-6, CRP, and enhances systemic inflammation present in OSA. Importantly, adipocytes have been identified to possess the ability produce IL-33, which in case of OSA patients seems crucial as the vast majority of them are obese, making adipose tissue an considerable source of the interleukin. However, the effect it exerts is not yet fully understood, and further research is needed, especially focusing on the mRNA expression of chosen genetic markers and animal models. Nevertheless, IL-33 through the intensification of TGF-β expression is involved in the stimulation of fibrosis and bronchial remodeling, including EMT, which might further contribute to the development and comorbidity of asthma and OSA.

The mechanisms of IL-33 activity described in this short review highlight its important role in the development of multidirectional inflammation. Therefore, direct or indirect blockade of this cytokine might greatly increase conservative therapy quality among patients with chronic respiratory diseases. At the moment, there are clinical trials involving anti-IL-33 antibodies which are very promising. Nonetheless, authors of this review indicate a necessity to undertake new research focusing on the development of peptide inhibitors for the ST2 receptors. The establishment of several interface regions between IL-33 and ST2 receptor, based on the available complex crystallographic structure, enables the creation of peptide library *in silico* through bioinformatics methods. Creation of peptide domain for the interface of IL-33 may be a universal method of safe, non-toxic and well-tolerated treatment for patients suffering from chronic inflammatory diseases of the respiratory system. At present, there is no investigation carried out in the direction suggested by the authors.

## Author Contributions

AG and MP created the concept of the paper. AG conducted the literature research and wrote the manuscript. PK, AA, PB, and MP revised the paper.

### Conflict of Interest Statement

The authors declare that the research was conducted in the absence of any commercial or financial relationships that could be construed as a potential conflict of interest.

## Abbreviations

AHI: apnea-hypopnea index
AREG: amphiregulin
BAL: bronchoalveolar lavage
COPD: chronic obstructive pulmonary sleep apnea
CRP: C-reactive protein
CXCL: chemokine (C-X-C motif) ligand
EMT: epithelial-to-mesenchymal transition
f-IL-33: full length interleukin-33
GAS: gamma interferon activation sites
GWS: genome-wide studies
IAK: interleukin-1 receptor associated kinase
IL: interleukin
IL-1Ra: interleukin 1 receptor antagonist
IL-1RacP: interleukin 1 receptor accessory protein
IL-36Ra: interleukin 36 receptor antagonist
ILC: innate lymphoid cell
ISRE: interferon stimulated response element
JNK: c-Jun N-terminal kinases
HF-HEV: nuclear factor from high endothelial venules
MAPK: mitogen-activated protein kinases
m-IL-33: mature interleukin 33
MHCII: major histocompatibility complex II
MYD88: myeloid differentiation primary response protein 88
NFκB: nuclear factor κB
OSA: obstructive sleep apnea
PDGF: platelet-derived growth factor
ROS: reactive oxygen species
SNP: single nucleotide polymorphism
sST2: suppression of tumorigenicity 2 soluble receptor
ST2: suppression of tumorigenicity 2 transmembrane receptor
TCR: Th2 cell receptor
TGF-β: transforming growth factor β
Th2: T helper 2 cell
TIR: Toll-like/interleukin 1receptor
TNF: tumor necrosis factor
TRAF6: tumor necrosis factor receptor-assosiatedreceptor-6.
